# Solution structure of the Equine Infectious Anemia Virus p9 protein: a rationalization of its different ALIX binding requirements compared to the analogous HIV-p6 protein

**DOI:** 10.1186/1472-6807-9-74

**Published:** 2009-12-17

**Authors:** Alok Sharma, Karsten Bruns, René Röder, Peter Henklein, Jörg Votteler, Victor Wray, Ulrich Schubert

**Affiliations:** 1 Department of Structural Biology, Helmholtz Centre for Infection Research, D-38124 Braunschweig, Germany; 2 Institute of Virology, Friedrich Alexander University of Erlangen-Nürnberg, D-91054 Erlangen, Germany; 3 Institute of Biochemistry, Charité-Universitätsmedizin-Berlin, D-10117 Berlin, Germany

## Abstract

**Background:**

The equine infection anemia virus (EIAV) p9 Gag protein contains the late (L-) domain required for efficient virus release of nascent virions from the cell membrane of infected cell.

**Results:**

In the present study the p9 protein and N- and C-terminal fragments (residues 1-21 and 22-51, respectively) were chemically synthesized and used for structural analyses. Circular dichroism and ^1^H-NMR spectroscopy provide the first molecular insight into the secondary structure and folding of this 51-amino acid protein under different solution conditions. Qualitative ^1^H-chemical shift and NOE data indicate that in a pure aqueous environment p9 favors an unstructured state. In its most structured state under hydrophobic conditions, p9 adopts a stable helical structure within the C-terminus. Quantitative NOE data further revealed that this α-helix extends from Ser-27 to Ser-48, while the N-terminal residues remain unstructured. The structural elements identified for p9 differ substantially from that of the functional homologous HIV-1 p6 protein.

**Conclusions:**

These structural differences are discussed in the context of the different types of L-domains regulating distinct cellular pathways in virus budding. EIAV p9 mediates virus release by recruiting the ALG2-interacting protein X (ALIX) via the YPDL-motif to the site of virus budding, the counterpart of the YPX_n_L-motif found in p6. However, p6 contains an additional PTAP L-domain that promotes HIV-1 release by binding to the tumor susceptibility gene 101 (Tsg101). The notion that structures found in p9 differ form that of p6 further support the idea that different mechanisms regulate binding of ALIX to primary versus secondary L-domains types.

## Background

Equine infectious anemia virus (EIAV) is a retrovirus of the lentivirus subfamily which also includes HIV-1, HIV-2 and simian immunodeficiency viruses (SIVs). Compared to the primate lentiviruses the EIAV genome is the smallest (~8.2 kb) and genetically simplest as it contains only three accessory genes (*rev*, *tat*, and *S2*) in addition to the canonical retroviral elements *gag*, *pol*, and *env*. As with other retroviruses the Gag polyprotein Pr55 of EIAV is required and sufficient for assembly and budding of virus like particles. The cleavage of the Pr55 Gag-precursor by the virus-encoded protease thereby yields the four major internal structural proteins: the matrix (MA, p15), capsid (CA, p26), nucleocapsid (NC, p11), and p9 proteins [1,2]. The Gag proteins are synthesized in the cytoplasm and targeted to the plasma membrane where they assemble into immature budding particles that consist predominantly of uncleaved polyproteins and are released from the cell membrane [3]. Maturation of the EIAV particle occurs concurrently with or shortly after release of the progeny virion in concert with protease activation.

The genomic position of p9 is analogous to that of the HIV-1 p6 protein and other similar proteins from different lentiviruses. Compared to HIV-1 p6, EIAV p9 has only minimal amino acid sequence homology and a considerable variation in the predicted secondary structure. Besides the function of p9 in viral DNA production and processing of the provirus [4], p9 plays, like p6 of HIV-1, an essential role in virus release, which are governed by late assembly domains (L-domains). Proline-rich L-domains, such as PTAP and PPPY have been identified in HIV-1, Rous sarcoma virus (RSV), and a variety of other enveloped viruses [5-7]. The HIV-1 PTAP motif specifically interacts with the N-terminus of the tumor susceptibility gene 101 (Tsg101), a component of the host endosomal sorting complex required for transport I (ESCRT I), a system that regulates membrane fission during multivesicular body (MVB) formation and cytokinesis [8-12]. Interestingly, the EIAV YPDL L-domain motif has been shown to interact with two cellular proteins, the ALG-2-interacting-protein-X (ALIX/AIP1; ALIX is used hereafter) [7,10] and the μ2 subunit of the AP-2 adaptor protein complex [13].

Although L-domains appear to interact with different cellular proteins, a certain functional interchangeability has been reported. For example, both PTAP and PPPY motifs can substitute for the YPDL domain to support EIAV replication [14]. These observations indicate that retroviruses, along with other enveloped viruses, have evolved different L-domains to specifically exploit certain host cellular machineries for virus budding and release.

Recently, we have characterized the structure of the HIV-1 p6 protein [15], and others have studied the structure of p6 fragments in complex with binding partners Tsg101 and ALIX [16,17]. Among known lentiviruses, the 51-amino acid EIAV p9 protein is one of the smallest proteins and the molecular structure has not been defined hitherto. With the goal of understanding the molecular mechanism involved in the biological function of p9, we have explored the high resolution structure and folding of p9, derived from the EIAV_WYOMING _isolate_, _under various solution conditions. Although the molecule exhibits a high degree of flexibility in a pure aqueous environment it adopts α-helical structures in an hydrophobic environment simulated by organic solvents. According to high resolution NMR data, p9 consists of two independent structural domains, an unstructured N-terminus and an extended C-terminal helix. The structure of p9 was compared with that of HIV-1 p6, and their similarities and differences are discussed in terms of differences in their L-domain functions.

## Results

### Synthesis and purification of synthetic p9 (sp9)

**Figure 1:**
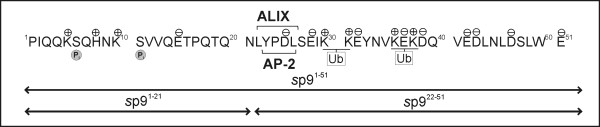
**Molecular characteristics of p9**. Primary amino acid sequence of the p9 protein is shown. Predicted phosphorylation sites, positions of positively and negatively charged side chains, sites for covalent attachment of ubiquitin (Ub) as well as the known binding domain for ALIX and AP-2 are indicated. Sequence positions of the synthetic peptides *s*p9 ^1-51^, *s*p9^1-21^, and *s*p9^22-51 ^are shown at the bottom of the figure.

An overview of the previously reported binding domains for ALIX and AP-2 within the EIAV p9 protein and their relationship to the primary structure derived from the EIAV_WYOMING _sequence, together with the predicted sites of post-translational modification, are shown in Fig. 1.

The *s*p9 molecule (*s*p9^1-51^) and its fragments (*s*p9^1-21 ^and *s*p9^22-51^) were chemically synthesized using solid-phase peptide synthesis (SPPS) and purified to homogeneity. The specific procedure, established previously by us for the HIV-1 p6 protein [15] with respect to the use of coupling agents, protection groups, cleavage reagents, and duration of coupling reactions, gave reproducibly high yields (usually 15%) of purified *s*p9^1-51^. It avoided problems normally encountered in such syntheses that include incomplete deprotection and coupling, inter- and intra-chain reaction with the resin matrix, side chain reactions, and peptide aggregation.

**Figure 2:**
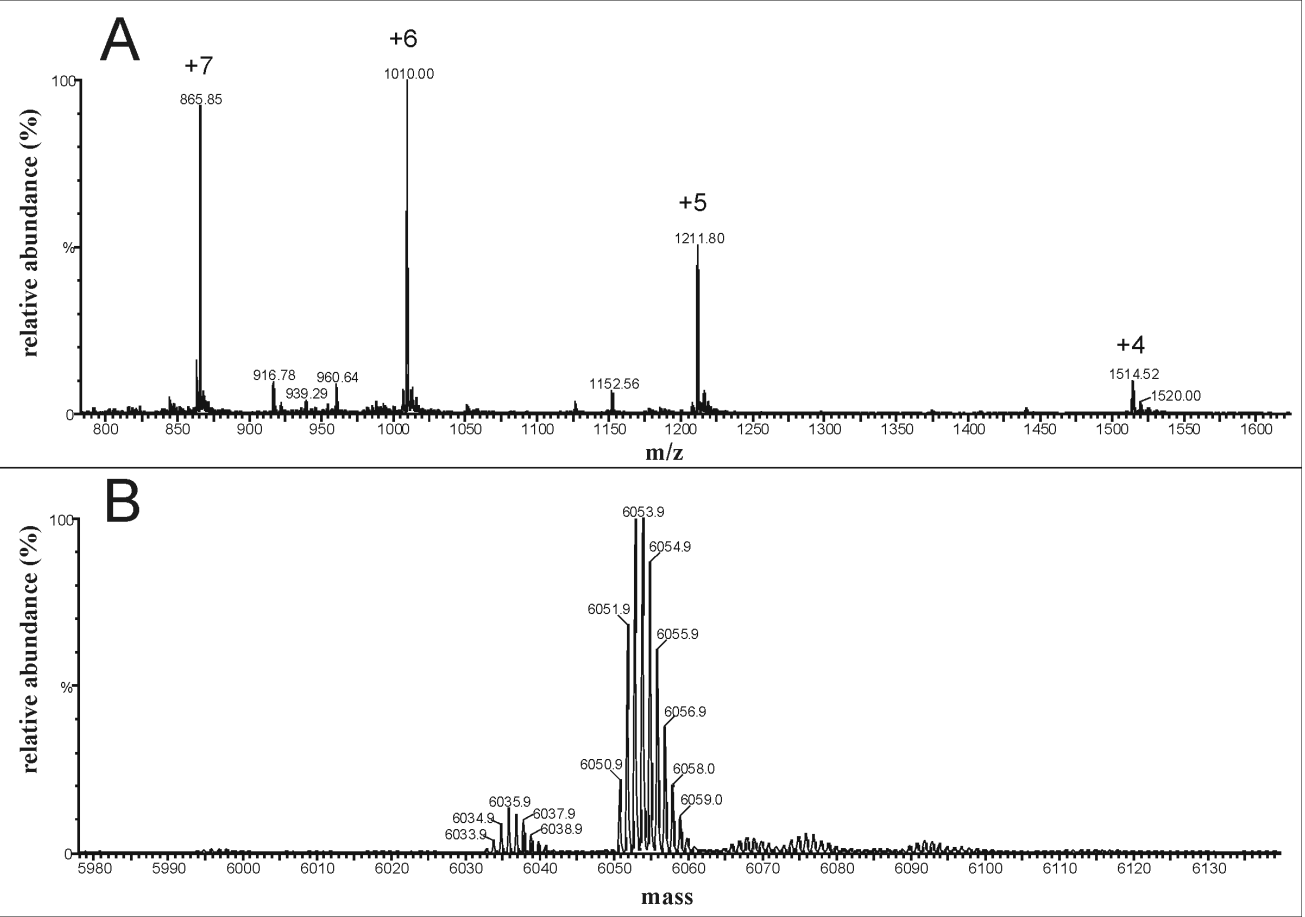
**Mass spectrum of *s*p9^1-51^**. A. Experimental mass spectrum of p9^1-51 ^showing the distribution of multiply charged ions, [M + 7H]^7+^, [M + 6H]^6+^, [M + 5H]^5+^, [M + 4H]^4+^. B. Deconvoluted mass spectrum showing the envelope of the molecular ion centred at 6053.9.

We also synthesized N- and C-terminal fragments of p9 using the same SPPS protocol. After cleavage from the resin, the crude peptides were purified. Illustrative data are shown in Additional file 1 for the full-length peptide *s*p9^1-51 ^and the N- and C-terminal fragments thereof (Additional file 1, Fig. S1-3). The purity of *s*p9^1-51 ^and its related fragment peptides was confirmed by molecular mass determination using positive ion electrospray ionization mass spectrometry (ESI-MS). The experimental results for *s*p9^1-51 ^showed a well defined multiply charged spectrum showing 7-4-fold positively charged ions (Fig. 2A) that was deconvoluted to give an intense envelope for the molecular ion cluster [M+H]^+ ^centered at a molecular mass of 6053.9 Da (Fig. 2B), corresponding to a calculated molecular weight of 6055.6 Da. This was confirmed by MALDI-MS (data not shown). Similarly, the N- and C-terminal fragments of p9 also afforded high quality data and the correct molecular masses (Additional file 1). The cumulative HPLC and MS data indicated *s*p9^1-51 ^and its fragments showed very little evidence of by-products and were pure enough (> 95%) for biophysical studies.

### Predicted structural details of EIAV p9

Several in-silico prediction programs have been employed to derive secondary structure information from the p9 primary sequence (EIAV_WYOMING_). All predictions converge to indicate that p9 is largely an unstructured molecule that has only a small propensity for helical structure (Table 1). The predicted helix is located in the C-terminal region, while the N-terminus of the molecule contains very little secondary structure if any. However, all the prediction algorithms indicate the C-terminal region has at least 7-residues in an α-helical conformation located between Leu-26 and Glu-32. A further short helix, approximately 5 residues in length, is centered on residue-42 (~residues Gln-40 to Leu-44). Thus, the *in silico *analysis suggests the existence of two C-terminal helices in p9.

**Table 1:** Secondary structure prediction for p9 using public domain semi-empirical programs

Method	α-helix 1	α-helix 2	Reference
Target99	26-33		[40]
SSpro	26-32	41-43	[41,42]
PORTER	26-33		[43]
PsiPred	26-33 (9-13)	41-42	[44]
PROF	26-32	36-39, 41-44	[45]
PHD	26-32		[46]
GOR I	25-51 (1-4)		[47]
GOR IV	26-36	40-44	[48]
HNN	25-32	40-44	[49]
SOPMA	24-33	41-51	[50]
GOR V	27-44		[51,52]

### C-terminus contributes to p9 secondary structure

A first insight into the secondary structure and folding of *s*p9^1-51 ^and its fragments thereof was obtained by analysis of the peptides at ambient temperature under various solution conditions by circular dichroism (CD) spectroscopy. We simulated a hydrophobic environment by using the organic solvent trifluoroethanol (TFE) to assess the degree of secondary structure under hydrophobic conditions. TFE is chosen for its well known characteristics as it favors intramolecular interactions and stabilizes secondary structure, particularly α-helices in domains of a peptide that have a propensity for such secondary structure [18]. As TFE tends to disrupt quaternary structure and dissociate peptide aggregates, it can alleviate problems occurring with intermolecular interactions in the higher concentration ranges required for NMR investigations and provide a platform to perform CD and NMR studies under similar solution conditions.

**Figure 3:**
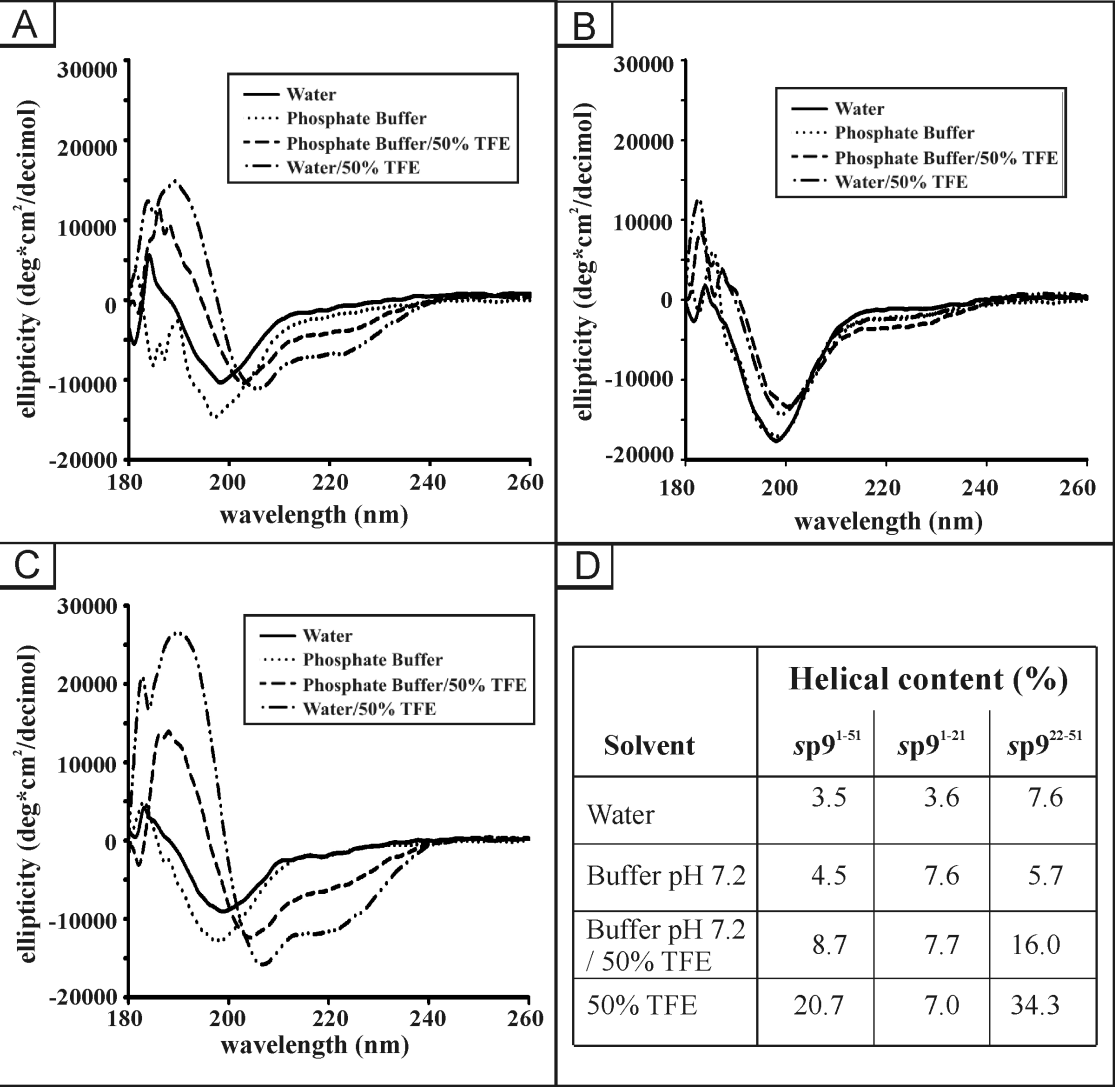
**Far UV CD spectra of *s*p9 and fragments thereof**. Far UV CD spectra of *s*p9^1-51 ^(A), *s*p9^1-21 ^(B), and *s*p9^22-51 ^(C) recorded at different TFE concentrations and different pH values. (D) Summary of secondary structure content (% helix) under different solution conditions.

The far-ultraviolet CD spectra of the full length molecule and its fragments are shown in Fig. 3. The spectrum of *s*p9^1-51 ^shows a strong ellipticity minimum at 196 nm with a small shoulder near 216 nm under aqueous (pure water, pH 3.0) conditions, which is characteristic of a random coil conformation with very little evidence of secondary structure (Fig. 3A). Although a negative signal near 200 nm could be associated with disordered structure, the broad nature of the band suggests the presence of some structure. A similar spectrum was obtained when the molecule was analyzed at physiological pH (phosphate buffer, pH 7.2). However, addition of 50% TFE (at pH 3), affords two sharp signals at 208 nm and 222 nm and a positive signal at ~189 nm, indicating establishment of an α-helical structure under these hydrophobic conditions. Yet, the full length molecule looses significant α-helical content in 50% TFE at neutral pH (Fig. 3A). Thus, the CD data suggest that *s*p9^1-51 ^adopts α-helical structure in the presence of a hydrophobic environment under acidic conditions that is less stable in neutral pH.

Two synthetic N- and C-terminal fragments were used to locate the structured region of the molecule by comparing the respective secondary structure contents. In 50% TFE (pH 3.0) the N- and C-terminal fragments showed ca. 7.0% and 34.3% helical content, respectively, indicating secondary structure is predominantly located in the C-terminal section of the molecule (Fig. 3B and 3C) whereas the N-terminal fragment *s*p9^1-21 ^showed no evidence of secondary structure under any of the conditions used, suggesting it is largely unstructured and does not contribute to the secondary structure of the molecule. In contrast, the C-terminal fragment exhibited folding behavior similar to that of *s*p9^1-51 ^under the same solution conditions (Fig. 3C and 3A). The percentage helical content calculated for the C-terminal fragment of ~34%, using the DICROPOT 2000 program, corresponds to 10 amino acids which is in-line with our observation for the full length molecule and confirms the C-terminus contains the locus for most of the secondary structure in the molecule (Table 2).

**Table 2:** Structural statistics for the 20 final structures of *s*p9^22-51 ^in 50% TFE

No. of distance constrains	
Total	358
Intraresidual (|*i*-*j*| = 0)	113
Sequential (|*i*-*j*| = 1)	137
Medium range (|*i*-*j*| ≤ 4)	108
Average number of NOE violations ≥ 0.2°A	0
Mean energies (kj/mol)	
*E*_total_	351.02
*E*_NOE_	95.98
*E*_van der Walls_	88.27
*E*_bond _+ *E*_angle _+ *E*_impropers_	167.1
Ramachandran plot^a^	
% residues with φ, ψ in most favourable regions	63
% residues with φ, ψ in additionally allowed regions	22.2
% residues with φ, ψ in generally allowed regions	14.8
% residues with φ, ψ in disallowed regions	0

^a ^Only for central structure

### Identification of structural elements in sp9^1-21^, sp9^22-51^, and sp9^1-51 ^by ^1^H NMR spectroscopy

In order to define in more detail the position of secondary structure identified by CD spectroscopy, we have recorded ^1^H NMR spectra of full length *s*p9^1-51 ^and the N- and C-terminal fragments *s*p9^1-21 ^and *s*p9^22-51 ^dissolved in 50% aqueous TFE-d_2_. Initially, we have analyzed the structural characteristics of the peptides on the basis of ^1^H_α _chemical shift data, which correlate with the chemical environment of the respective amino acid residues and therefore have proven to be useful for determining the presence, nature and exact position of secondary structure elements in such molecules [19]. For instance, a minimum of four adjacent residues, showing pronounced upfield shifts relative to random coil values (< -0.1 ppm) indicate local helical structure while downfield shifts (> 0.1 ppm) of three or more adjacent residues are indicative of α-helical structures. In order to obtain these data a set of one- and two-dimensional (1D, 2D) ^1^H NMR spectra was recorded for each peptide. Signal assignments of the NMR spectra were accomplished using a standard procedure combining homonuclear 2D TOCSY and 2D NOESY NMR spectral data [20]. Individual spin systems were identified from 2D TOCSY spectra, starting from the backbone amide protons. Sequence-specific assignments were determined from cross-peaks in the 2D NOESY spectra based on short observable distances between ^1^H_N_, ^1^H_α _and ^1^H_β _nuclei of amino acid residue i and ^1^H_N _of residue i+1. Spin systems that could readily be recognized were used as starting points to establish residue positions in the peptide sequence.

**Figure 4:**
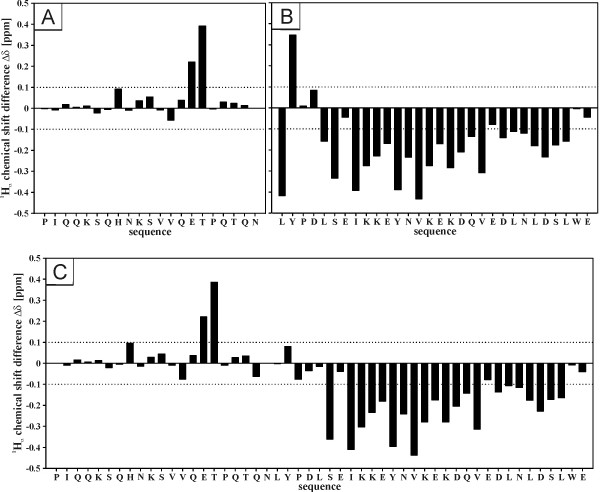
**Chemical shift differences of α-protons**. Chemical shift differences (ppm) of the α-protons between the experimental values and those for residues in a random coil for (A) *s*p9^1-21^, (B) *s*p9^22-51 ^(*B*), and (C) *s*p9^1-51 ^in 50% TFE at 300 K.

For each peptide the ^1^H_α _chemical shift differences relative to random coil values were determined and plotted against the respective sequence (Fig. 4A, B, and 4C). It can readily be seen that no substantial secondary structure is present in the N-terminal portion of *s*p9 (Fig. 4A and 4C) as no stretch of adjacent residues showing either upfield or downfield shifts is present. The only pronounced downfield shifts observed for Glu-15 and Thr-16 can be explained by the presence of a proline residue in position 17. As demonstrated previously in the context of HIV-1 Vpr [21,22] proline residues generally cause unusual intrinsic downfield shifts of 0.28 ppm ± 0.1 ppm in the preceding residues and of 0.08 ppm ± 0.03 ppm in residues two positions towards the N-terminus. Taking this proline-effect into consideration clearly rationalizes the downfield shifts of Glu-15 and Thr-16 and implies that these residues are in an unstructured environment.

Unlike the N-terminus, the C-terminal region shows a large number of upfield shifts comprising residues Ser-27 to Leu-49 and therefore is clearly indicative of the presence of helical structure in this section of the molecule (Fig. 4B and 4C). Even though residues Glu-28 and Glu-42 exhibit upfield shifts of only - 0.042 and - 0.079, respectively, it seems most likely that they are both part of a continuous helix, which in the case of Glu-28 becomes evident by the presence of very pronounced upfield shifts of the neighboring residues Ser-27 and Ile-29, and in the case of Glu-42 by a further stretch of seven weakly helical residues located directly to the C-terminal side of Glu-42. However, as the upfield shifts of these residues (Glu-42/Asp-43 to Leu-49) are distinctly less pronounced we assume that this part of the helix possesses decreased stability. At its N-terminus the helix is delimited by Pro-24, a residue that is often considered to be a helix-breaker.

A comparison of the ^1^H_α _chemical shift differences of the full length molecule with those of the two fragments reveals that they are almost identical apart from residues Leu-22 to Leu-26. This observation can easily be explained with the fact that this site represents the interface of the two fragments and therefore the respective C- (*s*p9^1-21^) or N-terminus (*s*p9^22-51^) whereas it is the central region of full length *s*p9.

**Figure 5:**
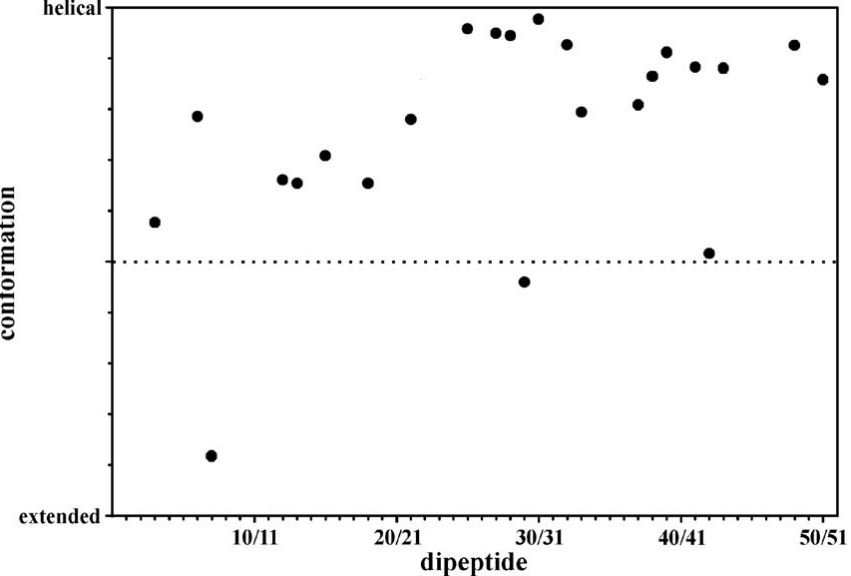
**Probabilities for helical, extended or random conformation in *s*p9 calculated from the ratios of the interproton distances *d*_NN _(i, i+1) and *dα*_N _(i, i+1) in the respective dipeptidic segments of *s*p^1-51 ^in 50% TFE at 300 K**.

It was possible to identify and quantify only a limited number of unambiguous medium range NOEs in the 2D NOESY spectrum of *s*p9^1-51 ^and these were insufficient to calculate a meaningful structure. However, they could be used to assess secondary structure in the full length molecule. The interproton distances *d*_NN_(i, i+1) and *d*_αN_(i, i+1) were determined and their ratios were used to calculate the probabilities for secondary structures in short segments (dipeptides) of the *s*p9^1-51 ^molecule. In an ideal α-helix *d*_NN_(i, i+1) is 2.8 Å and *d*_αN_(i, i+1) is 3.5 Å while an extended strand shows distances of 4.3 Å for *d*_NN_(i, i+1) and of 2.2 Å for *d*_αN_(i, i+1), respectively. Using the equation given by Bradley *et al*. [23] the combination of these and the experimentally determined values of these distances allows an estimation of whether the respective dipeptides are in a helical, extended or a more unordered conformations. Fig. 5 shows the probability for particular secondary structure against the sequence of *s*p9. Although there was no full sequence coverage with the required signals from the NOESY spectrum it can readily be deduced from the plot that no marked and well defined secondary structure is present in the N-terminal section of the molecule. Even though most dipeptides in the region Pro-1/Ile-2 to Asn-21/Leu-22 show a weak propensity for helix formation the merely moderate increase of values suggest a more random rather than a stable helical conformation. These change towards the C-terminus (Asp-25/Leu-26 to Trp-50/Glu-51) where a large majority of the observed distance combinations results in clearly increased values thereby implying the presence of an α-helix in this region.

### Structure calculations from quantitative NOE data

The problem observed above for the full length molecule was caused by the overlap of signals that could therefore not be unambiguously identified or accurately quantified in the 2D spectra. Most likely, this problem arose from the high proportion of similar amino acids in the p9 sequence (Asn/Asp, Gln/Glu and Leu/Ile) that are distributed throughout the molecule. As all the qualitative data indicate the sole structured region in the molecule is restricted to the C-terminal region we focused our attention on the C-terminal peptide. This resolved the problem of signal overlap and allowed identification of sufficient medium range NOEs for structure calculations.

**Figure 6:**
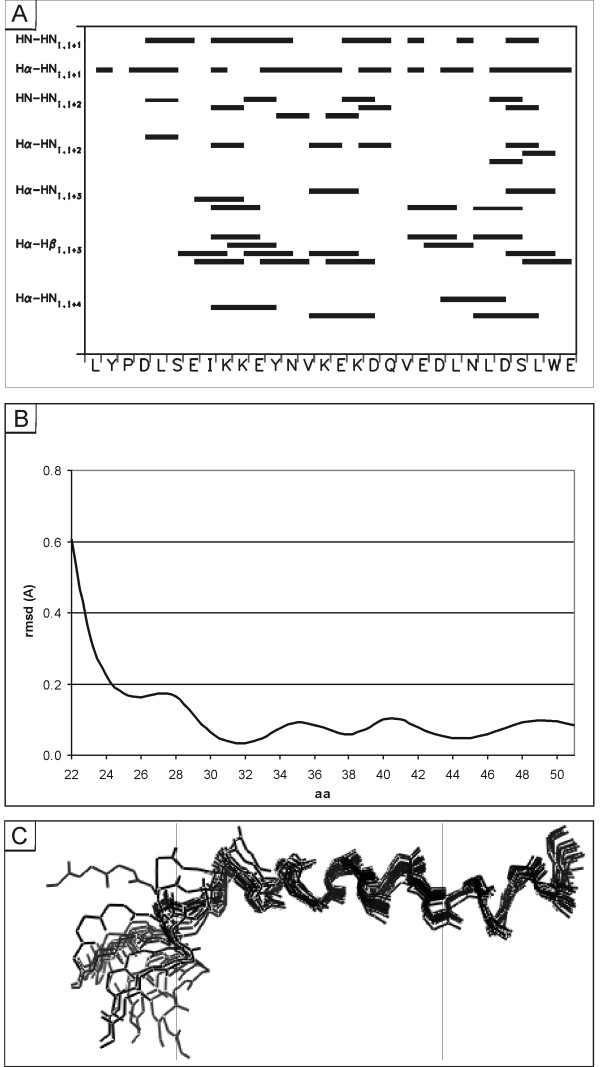
**Summary of the observed NOEs for the C-terminus of *s*p9**. (A) Summary of the observed short and medium range NOEs for *s*p9^22-51 ^in 50% TFE at 300 K. (B) Root mean square deviations (rmsd) for the backbone atoms of *s*p9^22-51 ^in each residue calculated using the consecutive segment method plotted against the residue number for the 20 final structures. (C) Supposition of the 20 best final restrained structures of *s*p9^22-51 ^after alignment of the backbone atoms of residues Ser-27 to Ser-48. Shown are structures comprising residues Leu-22 to Glu-51.

Hence after quantification of the NOE data a total of 346 NOEs (Fig. 6A and Table 2) were used as distance restraints to calculate 100 structures using a standard protocol [24]. The 20 structures with the lowest NOE and total energies and without distance violations greater than 0.2 Å were chosen for the final fitting analysis (Table 2). The heterogeneity within these structures was assessed using the consecutive segment approach, in which the rmsd (root mean square deviations) of the backbone atoms for short segments, 2-5 residues in length, were systematically and pair wise determined [25]. This analysis allows identification of regions of high similarity within the 20 final conformations and therefore identification of stable structural elements. The best defined regions of the molecule were then those showing rmsd of the backbone atoms of less than 0.2 Å, namely a continuous stretch comprising amino acid residues Asp-25 to Glu-51 in which the 20 refined structures share a high degree of similarity (Fig. 6B). This finding is in good agreement with the ^1^H_α _chemical shift data, the observed qualitative NOEs, and the Bradley-analysis which all suggest the presence of a well defined α-helix in the region Ser-27 to Leu-49.

**Figure 7:**
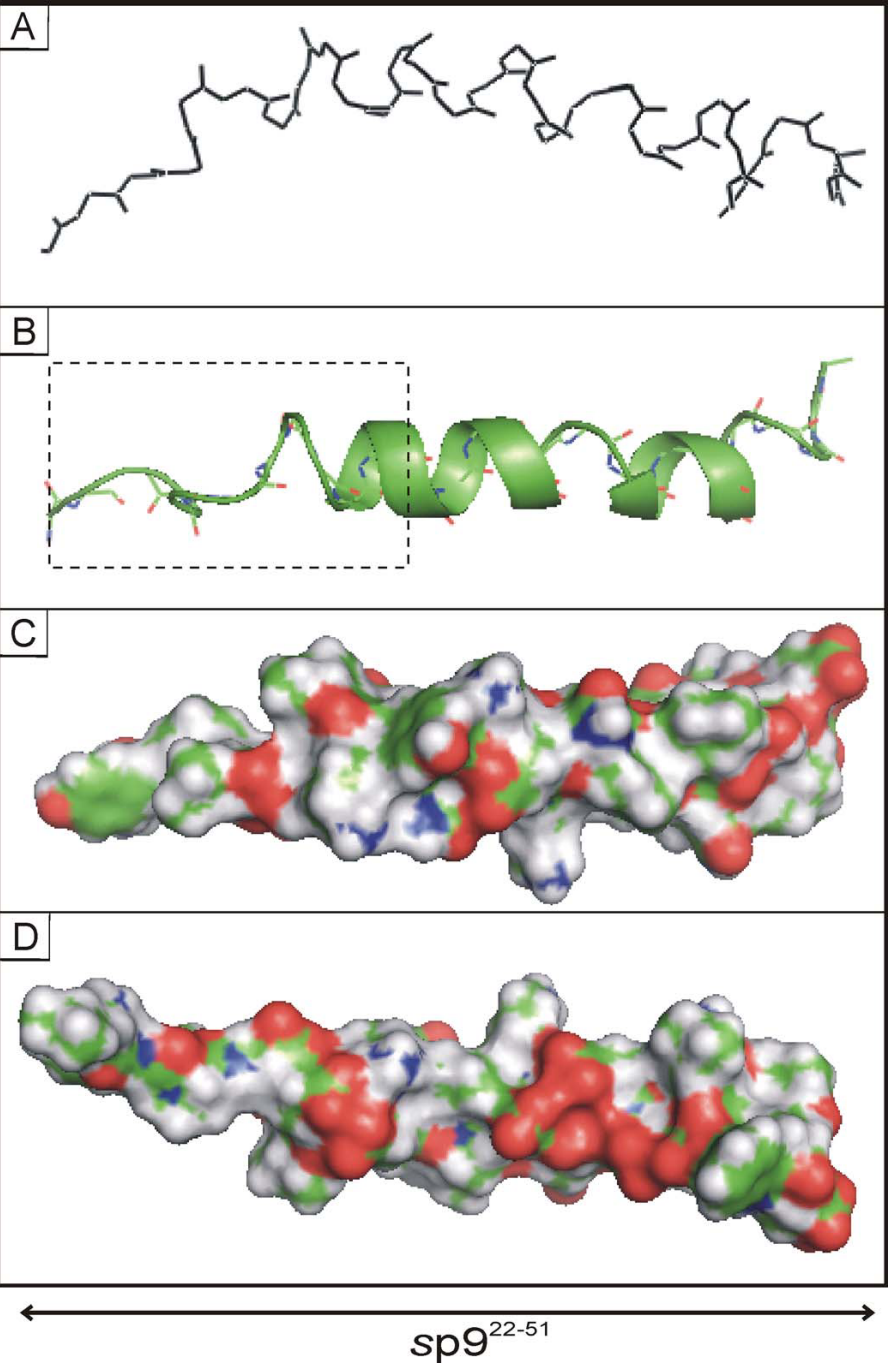
**Final structure of *s*p9^22-51 ^in 50% TFE**. (A) Central structure of *s*p9^22-51 ^that shows the lowest average rmsd value to all other final structures. (B) Ribbon plot of the same showing helical regions. The boxed area corresponds to the position of the ALIX-binding domain. (C) Space-filling representation of the central structure in the same orientation as in A. (D) Space-filling representation rotated 180°.

A central structure, in terms of the position in 3D space, was determined for the selected 20 lowest NOE and total energy structures using LSQMAN and MOLMAN2 (Uppsala Factory Package [26]). The central structure was then used as a template to superimpose and compare the other 19 refined low energy structures and the resulting set of aligned conformations is shown in Fig. 6C. The central structure is shown in Fig. 7 with a helical conformation between residues Ser-27 and Leu-49.

## Discussion

### Secondary structure of p9 is strongly dependent upon solution conditions

Previous studies have established p9 as the functional equivalent of the HIV-1 p6 protein, although these proteins of almost identical size have only limited sequence homology. Therefore, a comparison of the structural properties of these two analogous proteins is required to understand their structure-function relationships and their interactions with the same cellular factors such as ALIX. In water, p6 adopts a random coil conformation without any preference for secondary structure [15,27] while in a hydrophobic environment specific regions, residues 14-18 (helix 1) and residues 35-44 (helix 2), of the molecule adopt helical structure [15]. Helix 2 of HIV-1 p6 appears to be important for specific binding interactions with ALIX [16].

In the quest to establish the structural details of the EIAV p9 molecule, CD analysis of *s*p9^1-51 ^shows that the molecule has no significant or very little secondary structure when dissolved in pure water at pH 3 or in buffer at physiological pH 7.2. However, secondary structure is observed and clearly stabilized in the presence of increasing amounts (up to 50%) of TFE used to introduce a more hydrophobic environment that is assumed to more closely simulate *in vivo *conditions where p9 is exposed to the hydrophobic surfaces of other proteins. However, for *s*p9^1-51 ^maximum α-helical content was found at room temperature in 50% aqueous TFE at pH 3 that decreased upon changing the pH to 7.2, irrespective of the hydrophobic environment (Fig. 3A). The CD data for the N- and C-terminal fragments clearly indicate secondary structure formation in solution is restricted to the C-terminus of the molecule. This fragment also behaves in a similar manner to the full length molecule in that maximal structure is found in 50% TFE and is less stable at physiological pH (Fig. 3C).

As in our previous structural elucidation of HIV-1 p6 we completely assigned the 1D and 2D ^1^H NMR spectra of *s*p9^1-51^, in conjunction with its N- and C-terminal fragments, to afford the position and nature of structured regions in the molecule. Well established criteria used previously by us, namely ^1^H_α _chemical shifts, indicate in its most structured state *s*p9 shows one region with a propensity for α-helical structure in the C-terminal region of the protein extending from Leu-26/Ile-28 to Ser-48. According to the chemical shift differences in both the full-length *s*p9 and the shorter C-terminal peptide *s*p9^22-51 ^there is some indication that the structured region extends back to residue Leu-26. The smaller negative differences observed towards the C-terminus indicate a weakening of the helical interactions in this region. A probability analysis for helical or extended conformation of dipeptidic segments for a limited number of interproton distances in the full length molecule support this conclusion (Fig. 5). The C-terminal fragment exhibited 34.3% helical content in 50% TFE. In contrast to the C-terminal peptide there was no evidence of any structured region in the N-terminus either in the full length molecule or its N-terminal peptide. This was apparent even under the most favorable conditions (50% TFE at pH 3) independently from both the CD and NMR data.

Based on the above and the limited number of unambiguously assigned medium range NOEs caused by signal overlap of similar amino acid spin systems in *s*p9^1-51^, structural calculations were undertaken of the C-terminal fragment *s*p9^22-51^, which contains the L-domain and the only structured region of the molecule. The molecular dynamic calculations, using a total of 358 quantitative NOEs that included 137 sequential and 108 medium range NOEs, afford a central arrangement that confines the helical structure to the region between Glu-28 to Asp-47 (Fig. 7).

### Comparison of the structure of EIAV p9 and HIV-1 p6

A considerable amount of literature now exists suggesting that EIAV p9 and HIV-1 p6 have several functions in common. Interestingly, these two functionally analogous proteins possess quite different biochemical and biophysical properties i.e. primary sequence, hydrophilicity and net charge, which would be expected to lead to different protein-protein interactions in the respective host cell system. Both proteins possess little sequence homology (only ~7% identity) and are predicted to differ significantly in their phosphorylation propensities. HIV-1 p6 was characterized as a largely phosphorylated protein [28]. Similarly, both molecules have two ubiquitinylation sites and were shown to become mono-ubiquitinylated, and in the case of p6, sumoylated, Fig. 1[29-31].

**Figure 8:**
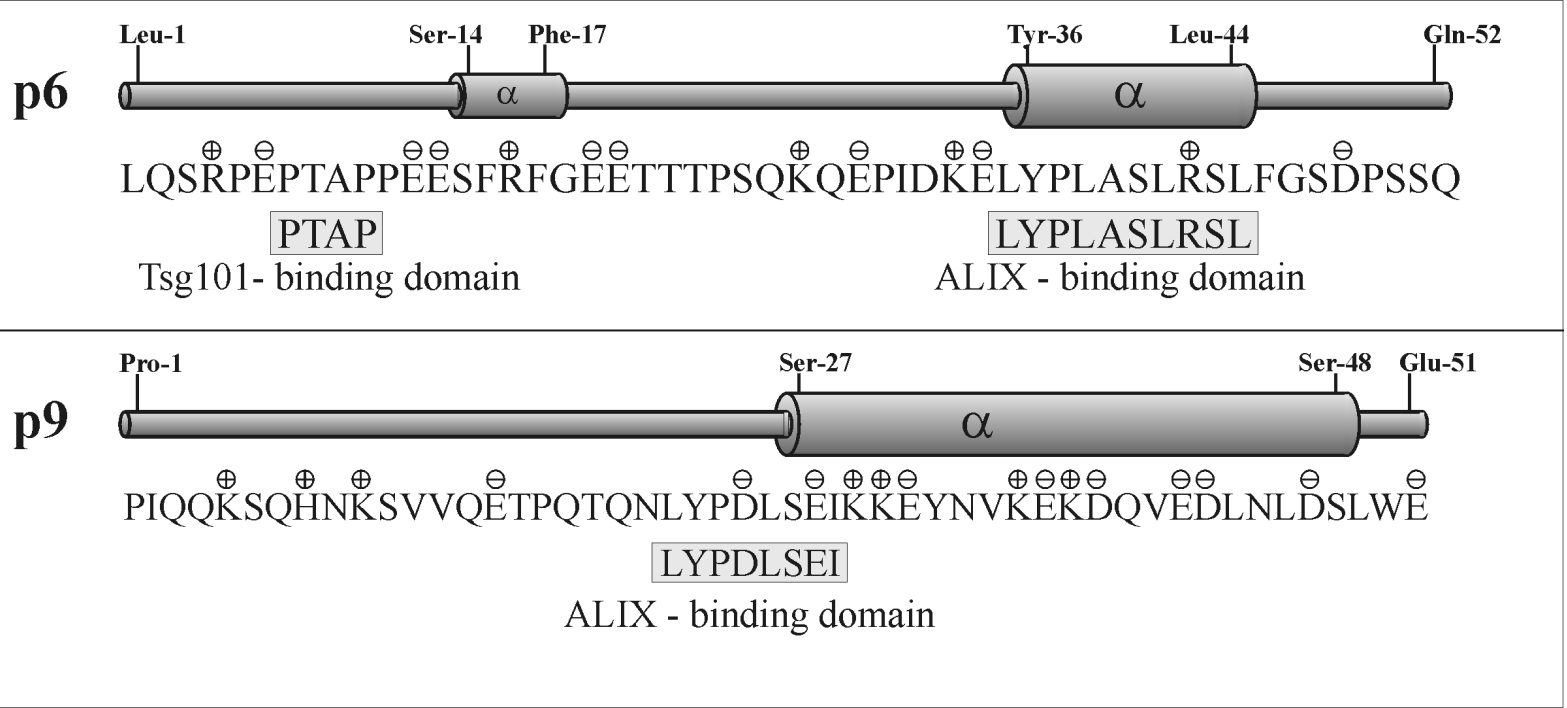
**Schematic comparison of the Tsg101- and ALIX-binding domains of p6 and p9 in relation to the experimentally determined secondary structures in 50% TFE**.

In their most structured states both molecules possess stable secondary structure although neither molecule possesses a stable tertiary structure. In a hydrophobic environment at low pH, both adopt helical secondary structure in their C-termini, although the helical region in p9 is longer (22 residues) than that of p6 (12 residues) under the same conditions. In each case the molecules are highly flexible and, unlike most structured proteins, must be considered as a dynamic equilibrium of many different conformers that have the overall propensity for secondary structure in the regions depicted in Fig. 8. Nevertheless, in its most structured form, the p6 molecule adopts a helix-turn-helix conformation in its C-terminal region whereas p9 assumes a single continuous helical conformation. The charge distribution within p6 and p9 is also distinctly different as the helix of p9 contains more charged residues than p6 (Fig. 8). Such differences in this helical region will be important for specific interactions with host cell factors (see below).

The L-domains of p9 and p6, as well as similar domains from other viral systems, have received considerable attention as they play critical roles in controlling the interaction with the host system that finally promotes viral budding and particle release [5-7]. These interactions involve the binding of specific regions of each molecule with components of the ESCRT [11,12]. Of particular relevance is the well-defined interaction of both molecules with ALIX, an ESCRT associated, multifunctional protein that interacts with both ESCRT-I and ESCRT-III. The ALIX-binding motif in both molecules responsible for this interaction has been defined recently as (L) [FY]PX_1-3_LXX [IL] [32-34] and corresponds in our case to ^22^**LYP**D**L**SE**I **in p9 and ^35^LYPLAS**L**RS**L **in p6 in which essential residues are in bold. Recently, the structure of ALIX has been worked out in detail [16,32,35] and its interaction with p6 was modeled based on NMR data of p6 [32]. In this model the C-terminal helix found in hydrophobic environments aligns coaxially with three helices in the ALIX V domain long arm to form a four-helix bundle [16]. The presence of a similar helical motif in the C-terminus of p9 presumably affords the same binding interaction. It is important to note that the ALIX-binding domain of p6 is located within the helical region of the molecule while in p9 it is located in a partially unstructured region.

The structural investigations on p6 and p9 offer a rationale for the different spacing of the essential residues in the ALIX-binding domains pointed out by Munshi *et al*. [33]. During ALIX binding hydrophobic conditions pertain and both molecules will adopt their most structured conformations. In p9 the ALIX-binding domain ^22^LYPDL^27^SEI motif is at the junction of an unstructured and beginning of the helical region (Ser-27) of the molecule in this conformation (Fig. 7 and 8), while in p6 the whole ^35^LYPLASLRSL motif is found within the helical region under the same conditions, Fig. 8[15]. In this helical conformation the essential lysine residue at position 42 in p6 is brought into the proximity of the 3 amino acid sequence LYP through the introduction of one helix turn, the intervening two residues are now on the opposite side of the helix away from the hydrophobic interaction site of the ALIX V domain [16]. This re-arranged recognition site of adjacent residues ^35^LYP ^40^SLRS**L **now imitates the ALIX motif LYPDLSEI of p9. Thus, the helical conformation in p6 is required to position the essential residues correctly and maintain the ALIX-p6 functional interaction by re-orienting the ^39^**LA **residues to the other side of helix away from the interaction site, while this is not necessary for p9. In addition, the C-terminal helix of p9 is substantially longer than the corresponding helix in p6 (Fig. 8) and thereby has a larger surface for potential interaction with the ALIX V domain. These structural differences in the ALIX-binding domains of the two molecules are reflected in the considerably higher thermodynamic stability of the p9-ALIX complexes [32,34] suggesting p9 has an optimized ALIX-binding site compared to that of p6 [34].

Recently, the X-ray structures of the complexes of ALIX with short synthetic peptides corresponding to the L-domains of HIV-1 p6 and EIAV p9 have been elucidated [34]. In these studies, the conserved tyrosine residue of the L-domains plays a crucial role in positioning the peptides in the same hydrophobic groove of arm 2 of the ALIX V domain. In keeping with our analysis of the solution structures an extra helical turn is observed in the crystal structure of p6 to position Leu-42 in the same position as Leu-26 in p9. These structures also define the orientation of the peptide chains and indicate the ALIX V domain is able to accommodate the short peptides without any major helical movements between the free and bound forms. According to our solution data the C-terminal helical region of both p6 and p9 could be accommodated towards the open neck of the ALIX V domain [16] while the flexible N-termini would allow these to be accommodated in the loop region. It remains to be determined whether these interactions of the full length proteins in the context of the uncleaved Gag polyprotein cause unfolding of ALIX.

Until recently the L-domains of p9 and p6 have usually been defined as those regions containing the YPDL and PT/SAP motifs, respectively [5-7]. Clearly this implies the ALIX-binding domain and L-domain motifs overlap (or are identical) in p9, but are separated in p6 where the L-domain is positioned proximal to the PTAP motif, Fig. 8.

For p6, the PTAP L-domain binds to Tsg101, the human equivalent of Vps23 of the yeast ESCRT-I complex that has recently been completely defined structurally [35]. Currently, there is no evidence of an equivalent interaction for p9. Indeed, the optimized ALIX binding site in p9 implies EIAV requires only a single L-domain for budding while the suboptimal binding site found in p6 requires the presence of a second site that functions through binding to Tsg101. This is corroborated by sequence data for p6 derived from different SIV strains where those containing high affinity ALIX binding sites do not have a Tsg101 binding site and vice versa [36].

Thus, it can be argued that the YPDL L-domain of EIAV does not require an independent ESCRT-I interaction [37,38] as the interactions between ESCRT-I and ESCRT-III are bridged through an appropriate conformation of ALIX stabilized by the strong interaction of p9. Presumably, in this bound form the N-terminal domain of ALIX binds to the ESCRT-III complex while the C-terminal proline-rich region binds the Tsg101 binding domain of ESCRT-I.

In summary, p9 from EIAV, like p6 from HIV-1, is structurally very labile and can exist in a number of conformational states that depend on its environment and the presence of binding partners that interact with specific domains in the molecule.

## Conclusions

Although p9 and p6 are sequentially quite different, both possess C-terminal helical structures in their most structured states that must be present during binding to the hydrophobic pocket of ALIX, a central control node in viral budding. Differences in the structural features in the vicinity of the ALIX-binding motif correlate with the different binding properties of the molecules and with the requirement of a further L-domain found only in p6 in the weakly structured N-terminal domain.

## Methods

### Peptides and protein

The sequence of full length p9 and its two fragments, p9^1-21 ^and p9^22-51^, used in this study is that derived from the isolate EIAV_WYOMING_, Fig. 1 (16).

### Peptide synthesis, purification, and characterization

The syntheses of the full length peptide were performed on an ABI 433A automated peptide synthesizer (Applied Biosystems, Darmstadt, Germany) on a 0.1 mM scale with 300 mg TentaGel S-Trt-Glu(tBu)-Fmoc-resin (capacity 0.17 mmol/g; RAPP Polymere GmbH Tübingen, Germany) using the Fmoc (*N*-(9-fluorenyl)methoxycarbonyl)/t-butyl strategy. The following side-chain protecting groups were used: t-butyloxycarbonyl (Trp, Lys), t-butyl ether (Thr, Ser, Tyr), t-butyl ester (Asp, Glu) and trityl (Asn, Gln and His). Couplings were performed with N- [1H-7-aza-benzotriazol(1-yl)(dimethylamino)-methylene]-N-methylmethanaminium hexafluoro-phosphate-N-oxide in N-methylpyrrolidone as coupling agent. Amino acids in positions 10 and 11 (Lys-Ser) were introduced as the pseudoproline derivative Fmoc-Lys(Boc)-Ser(Ψ^Me, Me ^Pro)-OH. Deprotection of the Fmoc group was performed during the complete synthesis with 20% piperidine in N,N-dimethylformamide. The final cleavage from the resin was performed with 95% TFA in water containing 3% triisopropylsilane and 5% phenol. The crude protein was purified by reversed phase HPLC (RP-HPLC) on a 7 μ Zorbax SB C18 column (21.2 × 250 mm) with a linear gradient of 50% B to 60% B in 45 min (A: 2500 ml water, 5 ml TFA; B: 2000 ml acetonitrile, 500 ml water, 5 ml TFA) at a flow rate of 10 ml/min with spectrophotometric monitoring at λ = 220 nm. The fractions were checked by RP-HPLC (Shimadzu LC10) on a Nulceosil C 18 column (4.6 × 125 mm, 5 μ, 300 Å) with a linear gradient of 10% B to 100% B over 45 min to give the final pure products. The fragments *s*p9^1-21 ^and *s*p9^22-51 ^were synthesized and purified in the same manner. The full length synthetic protein and the N- and C-terminal peptides are designated as *s*p9, *s*p9^1-21 ^and *s*p9^22-51^, respectively.

### Peptide sequencing and mass spectrometry

For *s*p9, the sequencing steps were completed on an Applied Biosystems 473A pulsed liquid phase sequencer according to a standard protocol. Positive ion ESI mass spectra were recorded on a Micromass Q-Tof-2™ mass spectrometer. Samples were dissolved in 70% aqueous methanol and infused at a flow rate of ca. 1 μl/min at ca. 0.8 kv needle voltage into the electrospray chamber The experimental spectra showing multiply charged molecular ions were deconvoluted with standard software. MALDI/TOF mass spectra were recorded on a Bruker reflex MALDI/TOF mass spectrometer using an N_2 _laser (337 nm) (see additional file 1).

### Circular dichroism (CD) spectroscopy

CD spectra of the protein samples of full-length *s*p9 and its related shorter fragments *s*p9^1-21 ^and *s*p9^22-51^were recorded at room temperature and a concentration of 0.2 mg ml^-1 ^in 0.5 mm cuvettes on a Jasco J-810 spectropolarimeter in a wavelength range from 260 to 180 nm at various pH values and trifluoroethanol (TFE) concentrations as described previously [15]. The resulting curves were smoothed using a high frequency filter, and secondary structure elements were quantified by deconvoluting the measured ellipticity using the DICROPROT 2000 program [39].

### ^1^H NMR spectroscopy

All one- (1D) and two-dimensional (2D) ^1^H NMR spectra of *s*p9 and its fragments *s*p9^1-21 ^and *s*p9^22-51 ^were recorded with (1D) or without (2D) spinning at 300 K on a Bruker Avance DMX 600 MHz instrument using a triple resonance probe head with gradient unit. The peptides were dissolved without pH adjustment (pH ~3.0) to final concentrations of 2-3 mM in 1:1 mixtures of H_2_O and CF_3_CD_2_OH (50% aqueous TFE-d_2_). Measurements were carried out with mixing times of 110 ms for the 2D TOCSY and 500 ms for the 2D NOESY experiments, respectively. Data acquisition, processing and spectral analysis were in all cases performed with standard Bruker software. All spectra were internally referenced to the residual TFE-H_2 _methylene signal at 3.95 ppm. The unambiguous amino acid spin systems and the sequential assignments (see additional file 1) were established using a standard procedure [15]. The complete signal assignments and ^1^H chemical shifts of *s*p9^22-51 ^have been deposited in the Biological Magnetic Resonance Data Bank under accession number RCSB100795.

### Structural calculations

The structure of *s*p9^22-51 ^was determined from quantitative NOE data as described in detail elsewhere [15]. Structures were calculated on a Silicon Graphics Octane work station using the program CNS 1.0 with standard CNS parameters for protein data sets [24]. A total of 346 distance restraints were used to generate 100 conformations of which 20 conformations, exhibiting no restraint violations greater than 0.2 Å and having the lowest energy values, were used for the final fitting analysis.

The heterogeneity within the final set of 20 structures was visualized using the consecutive segment approach which allows fitting regions for alignments to be defined (19). The central structure showing the lowest root mean square deviation (rmsd) of its fitting region to those of the other structures was then determined using the programs LSQMAN and MOLEMAN2 (Uppsala Software Factory) [26]. Finally, alignments were performed by superimposing the fitting regions of all other structures to that of the central structure and these were visualized with the PYMOL program http://www.pymol.org. The final structure of *s*p9^22-51 ^has been deposited in the Protein Data Bank under code PDBID 2K84.

In this study the probability for helical or extended conformation of dipeptidic segments in the full length *s*p9 molecule was analyzed using the distances between ^1^H nuclei of adjacent residues, namely H_N _and H_α _of residue i and H_N _of residue i+1 (*d*_NN_(i, i+1), *d*_αN_(i, i+1)) (22). The distances *d *which strictly correlate with signal intensities I (I ~1/*d*^6^) were obtained by transferring the intensities of the respective NOE signals into interproton distances using the Bruker program AURELIA. Only unambiguous signals were used for this analysis. For a few signals that were weakened by the pre-saturation of the water resonance a correction was applied (-1.5 Å when within 0.005 ppm of the water signal, -1 Å when within 0.025 ppm, and -0.5 Å when within 0.05 ppm), and a similar correction was made in cases where two or more signals could not be resolved individually due to close signal overlap. An equation given by Bradley *et al*. [23] was then used to calculate probabilities for helical or extended conformations.

## Authors' contributions

All authors read and approved the final manuscript. AS, KB, and VW planned and performed the structural analysis. RR and PH synthesized the peptides. JV, VW and US planned experiments and wrote the manuscript.

## Supplementary Material

Additional file 1 **Tables of ^1^H Chemical shifts of the full length and N- and C-terminal fragments of p9.** HPLC and MS data for the full length and N- and C-terminal fragments of p9. Click here for file
