# Supplementary material for: Solution structure of the Equine Infectious Anemia Virus p9 protein: a rationalization of its different ALIX binding requirements compared to the analogous HIV-p6 protein
Source: BMC Struct Biol. 2009 Dec 17;9:74. doi: 10.1186/1472-6807-9-74 (PMC2803184; doi:10.1186/1472-6807-9-74)
Supplement: Additional file 1 — Tables of 1H Chemical shifts of the full length and N- and C-terminal fragments of p9. HPLC and MS data for the full length and N- and C-terminal fragments of p9. [file 1472-6807-9-74-S1.DOC]

**Additional File for:**

# **Solution structure of the Equine Infectious Anemia Virus p9 protein: a rationalization of its different ALIX binding requirements compared to the analogous HIV-p6 protein**

# Alok Sharma^1,2*^, Karsten Bruns^1,2*^ , René Röder^2,3^ , Peter Henklein^3^, Jörg Votteler^2^, Victor Wray^1^, and Ulrich Schubert^2^

^1^ Department of Structural Biology, Helmholtz Centre for Infection Research, D-38124 Braunschweig, Germany

^2^ Institute of Virology, Friedrich Alexander University of Erlangen-Nürnberg, D-91054 Erlangen, Germany

^3^ Institute of Biochemistry, Charité-Universitätsmedizin-Berlin, D-10117 Berlin, Germany

^*^ These contributed equally to the work

**Correspondence**

Dr. Victor Wray, Department of Structural Biology, Helmholtz Centre for Infection Research, Inhoffenstraße 7, D-38124 Braunschweig, Germany. Tel.: +49-(0)531-6181-7200. Fax: +49-(0)531-6181-7099. E-mail: victor.wray@helmholtz-hzi.de

**TABLES OF ^1^H Chemical shifts of the full length and N- and C-terminal fragments of p9.**

**Table 1.** ^1^H chemical shifts for *s*p9^1-21^ in 50% TFE at 300 K.

|  | HN | **Hα** | **Hβ** | **Hγ** | **Hδ** | **Hε** | **Hζ** | **Hη** |
| --- | --- | --- | --- | --- | --- | --- | --- | --- |
| **P** |  | 4.438 | 2.375/1.974 | 2.042 (2x) | 3.87/3.792 |  |  |  |
| **I** | 8.483 | 4.222 | 1.897 | γ1: 1.553/1.251; γ2: 0.972 (3x) | 0.972 (3x) |  |  |  |
| **Q** | 8.483 | 4.388 | 2.121/2.034 | 2.422 (2x) |  | * |  |  |
| **Q** | 8.361 | 4.374 | 2.173/2.042 | 2.423 (2x) |  | * |  |  |
| **K** | 8.294 | 4.371 | 1.935/1.838 | 1.512 (2x) | 1.751 (2x) | 3.042 (2x) | n. d. |  |
| **S** | 8.146 | 4.478 | 3.958/3.89 |  |  |  |  |  |
| **Q** | 8.282 | 4.364 | 2.13/2.002 | 2.386 (2x) |  | * |  |  |
| **H** | 8.439 | 4.721 | 3.333/3.205 |  | δ2: 7.334 | ε1: 8.595 |  |  |
| **N** | 8.346 | 4.74 | 2.892/2.845 |  | δ2: 7.544/6.85 |  |  |  |
| **K** | 8.258 | 4.396 | 1.929/1.873 | 1.508 (2x) | 1.75 (2x) | 3.049 (2x) | n. d. |  |
| **S** | 8.209 | 4.554 | 3.948/3.909 |  |  |  |  |  |
| **V** | 7.957 | 4.173 | 2.188 | γ1: 0.995 (3x); γ2: 0.995 (3x) |  |  |  |  |
| **V** | 7.804 | 4.124 | 2.12 | γ1: 0.985 (3x); γ2: 0.985 (3x) |  |  |  |  |
| **Q** | 8.136 | 4.408 | 2.161/2.062 | 2.395 (2x) |  | * |  |  |
| **E** | 8.161 | 4.51 | 2.18/2.034 | 2.493 (2x) |  |  |  |  |
| **T** | 7.984 | 4.742 | 4.373 | γ2: 1.298 (3x) |  |  |  |  |
| **P** |  | 4.437 | 2.375/1.974 | 2.042 (2x) | 3.87/3.792 |  |  |  |
| **Q** | 8.341 | 4.4 | 2.2/2.072 | 2.475 (2x) |  | * |  |  |
| **T** | 7.955 | 4.373 | 4.373 | γ2: 1.259 (3x) |  |  |  |  |
| **Q** | 8.238 | 4.383 | 2.188/2.071 | 2.414 (2x) |  | * |  |  |
| **N** | 8.243 | 4.75 | 2.853/2.785 |  | δ2: 7.544/6.85 |  |  |  |

Footnotes: n. d.: not determined, * all Hε21- and Hε22-resonance signals were found at 7.583-7.402 and at 6.948-6.65, respectively, but could not be assigned specifically.

**Table 2.** ^1^H chemical shifts for *s*p9^22-51^ in 50% TFE at 300 K.

|  | **HN** | **Hα** | **Hβ** | **Hγ** | **Hδ** | **Hε** | **Hζ** | **Hη** |
| --- | --- | --- | --- | --- | --- | --- | --- | --- |
| L |  | 3.962 | 1.714 (2x) | 1.646 | 0.968 (2x3x) |  |  |  |
| Y | 8.225 | 4.946 | 3.161/2.946 |  | 7.22 (2x) | 6.898 (2x) |  |  |
| **P** |  | 4.448 | 2.226/1.938 | 2.036 (2x) | 3.783/3.561 |  |  |  |
| **D** | 8.005 | 4.844 | 3.036/2.889 |  |  |  |  |  |
| **L** | 8.208 | 4.222 | 1.759 (2x) | 1.759 | 1.002 (3x)/0.924 (3x) |  |  |  |
| **S** | 8.31 | 4.166 | 4.019 (2x) |  |  |  |  |  |
| **E** | 7.756 | 4.245 | 2.214 | 2.552 (2x) |  |  |  |  |
| **I** | 7.745 | 3.838 | 2.019 | γ1: 1.713/1.25 γ2: 0.968 (3x) | 0.89 (3x) |  |  |  |
| **K** | 8.146 | 4.086 | 1.94/1.724 | 1.482 (2x) | 1.626 (2x) | 2.991 (2x) | 7.613 (3x) |  |
| **K** | 7.847 | 4.132 | 2.028/1.763 | 1.467 (2x) | 1.67 (2x) | 3.002 (2x) | 7.596 (3x) |  |
| **E** | 8.203 | 4.121 | 2.291/2.201 | 2.664/2.472 |  |  |  |  |
| **Y** | 8.592 | 4.211 | 3.172 (2x) |  | 7.151 (2x) | 6.818 (2x) |  |  |
| **N** | 8.166 | 4.516 | 3.06/2.864 |  | 7.474/6.801 |  |  |  |
| **V** | 8.118 | 3.748 | 2.245 | 1.126 (3x)/1.014 (3x) |  |  |  |  |
| **K** | 8.146 | 4.086 | 1.941/1.709 | 1.478 (2x) | 1.613 (2x) | 3.01 (2x) | 7.595 (3x) |  |
| **E** | 8.265 | 4.119 | 2.082 (2x) | 2.405/2.33 |  |  |  |  |
| **K** | 7.982 | 4.076 | 1.985/1.736 | 1.515 (2x) | 1.59 (2x) | 3.037 (2x) | 7.575 (3x) |  |
| **D** | 8.361 | 4.551 | 3.047/2.945 |  |  |  |  |  |
| **Q** | 8.073 | 4.234 | 2.246 (2x) | 2.506/2.427 |  | 7.129/6.672 |  |  |
| **V** | 8.05 | 3.872 | 2.239 | 1.093 (3x)/1.002 (3x) |  |  |  |  |
| **E** | 8.265 | 4.211 | 2.213 (2x) | 2.63/2.518 |  |  |  |  |
| **D** | 8.214 | 4.618 | 3.038/2.972 |  |  |  |  |  |
| **L** | 8.011 | 4.268 | 1.862/1.798 | 1.658 | 0.946 (2x3x) |  |  |  |
| **N** | 8.146 | 4.63 | 2.878 (2x) |  | 7.417/6.621 |  |  |  |
| **L** | 8.163 | 4.2 | 1.782 (2x) | 1.601 | 0.978 (3x)/0.89 (3x) |  |  |  |
| **D** | 8.27 | 4.527 | 2.978/2.926 |  |  |  |  |  |
| **S** | 7.954 | 4.324 | 3.985/3.934 |  |  |  |  |  |
| **L** | 7.745 | 4.222 | 1.672 (2x) | 1.48 | 0.915 (3x)/0.835 (3x) |  |  |  |
| **W** | 7.694 | 4.697 | 3.364 (2x) |  | δ1: 7.265 | ε1: 9.683; ε3: 7.632 | ζ2: 7.457; ζ3: 7.219 | η2: 7.152 |
| **E** | 7.768 | 4.245 | 2.114/1.942 | 2.303 (2x) |  |  |  |  |

**Table 3.** ^1^H resonance assignments for *s*p9^1-51^ in 50% TFE at 300 K.

| residue | NH | H^α^ | H^β^ | other |
| --- | --- | --- | --- | --- |
| P1 |  | 4.44 | 2.554, 2.126 | H^γ^, * 2.126, 2.126; H^δ^, * 3.482, 3.424 |
| I2 | 8.49 | 4.221 | 1.9 | H^γ1^, * 1.553, 1.255; H^γ2^, * 0.973; H^δ1^, * 0.973 |
| Q3 | 8.484 | 4.386 | 2.113, 2.022 | H^γ^, * 2.422, 2.422 ^##^ |
| Q4 | 8.355 | 4.375 | 2.172, 2.043 | H^γ^, * 2.423, 2.423 ^##^ |
| K5 | 8.287 | 4.373 | 1.938, 1.846 | H^γ^, * 1.514, 1.514; H^δ^, * 1.752, 1.752; H^ε^, * 3.04, 3.04; N^ζ^, * 7.602 |
| S6 | 8.14 | 4.479 | 3.957, 3.886 |  |
| Q7 | 8.27 | 4.366 | 2.137, 1.998 | H^γ^, * 2.389, 2.389 ^##^ |
| H8 | 8.434 | 4.725 | 3.336, 3.208 | H^δ2^, * 7.332; H^ε1^, * 8.597 |
| N9 | 8.349 | 4.736 | 2.862, 2.862 | N^δ2^, * 7.535, 6.841 |
| K10 | 8.247 | 4.388 | 1.933, 1.834 | H^γ^, * 1.5, 1.5; H^δ^, * 1.742, 1.742; H^ε^, * 3.04, 3.04; N^ζ^, * 7.602 |
| S11 | 8.199 | 4.543 | 3.931, 3.931 |  |
| V12 | 7.948 | 4.171 | 2.181 | H^γ1^, * 0.989; H^γ2^, * 0.989 |
| V13 | 7.801 | 4.105 | 2.113 | H^γ1^, * 0.984; H^γ2^, * 0.984 |
| Q14 | 8.129 | 4.407 | 2.169, 2.044 | H^γ^, * 2.399, 2.399 ^##^ |
| E15 | 8.134 | 4.511 | 2.176, 2.028 | H^γ^, * 2.491, 2.491 |
| T16 | 7.981 | 4.736 | 4.386 | H^γ2^, * 1.3 |
| P17 |  | 4.431 | 2.367, 1.963 | H^γ^, * 2.136, 2.136; H^δ^, * 3.875, 3.796 |
| Q18 | 8.388 | 4.397 | 2.214, 2.077 | H^γ^, * 2.491, 2.491 ^##^ |
| T19 | 7.954 | 4.385 | 4.295 | H^γ2^, * 1.276 |
| Q20 | 8.224 | 4.307 | 2.156, 2.077 | H^γ^, * 2.424, 2.424 ^##^ |
| N21 | 8.164 | 4.75 | 2.884, 2.79 | N^δ2^, * 7.446, 6.71 |
| L22 | 7.852 | 4.378 | 1.64, 1.64 | H^γ^, * 1.605; H^δ1^, * 0.941; H^δ2^, * 0.87 |
| Y23 | 7.953 | 4.679 | 3.155, 3.073 | H^δ1^, * 7.147; H^δ2^, * 7.147; H^ε1^, * 6.86; H^ε2^, * 6.86 |
| P24 |  | 4.364 | 2.333, 1.92 | H^γ^, * 2.148, 2.033; H^δ^, * 3.811, 3.672 |
| D25 | 7.885 | 4.725 | 3.008, 2.953 |  |
| L26 | 8.281 | 4.365 | §§ | ^§§^ H^δ1^, * 0.939; H^δ2^, * 0.939 |
| S27 | 8.292 | 4.139 | 4, 3.896 |  |
| E28 | 7.716 | 4.251 | 2.112, 1.943 | H^γ^, * 2.575, 2.311 |
| I29 | 7.914 | 3.82 | 2.023 | H^γ1^, * 1.76, 1.237; H^γ2^, * 0.959; H^δ1^, * 0.881 |
| K30 | 8.185 | 4.057 | 1.937, 1.844 | H^γ^, * 1.522, 1.481; H^δ^, * 1.742, 1.742; H^ε^, * 3.041, 3.041; N^ζ^, * 7.602 |
| K31 | 7.874 | 4.126 | 2.04, 1.763 | H^γ^, * 1.466, 1.466; H^δ^, * 1.673, 1.673; H^ε^, * 2.995, 2.995; N^ζ^, * 7.602 |
| E32 | 8.257 | 4.11 | 2.311, 2.211 | H^γ^, * 2.672, 2.492 |
| Y33 | 8.648 | 4.205 | 3.187, 3.187 | H^δ1^, * 7.154; H^δ2^, * 7.154; H^ε1^, * 6.814; H^ε2^, * 6.814 |
| N34 | 8.168 | 4.51 | 3.073, 2.869 | N^δ2^, * 7.482, 6.811 |
| V35 | 8.134 | 3.742 | 2.26 | H^γ1^, * 1.121; H^γ2^, * 1.016 |
| K36 | 8.145 | 4.081 | 1.915, 1.706 | H^γ^, * 1.463, 1.463; H^δ^, * 1.605, 1.605; H^ε^, * 2.998, 2.998; N^ζ^, * 7.602 |
| E37 | 8.264 | 4.115 | 2.4, 2.111 | H^γ^, * 2.493, 2.493 |
| K38 | 7.987 | 4.081 | 1.983, 1.706 | H^γ^, * 1.511, 1.511; H^δ^, * 1.601, 1.601; H^ε^, * 3.035, 3.035; N^ζ^, * 7.602 |
| D39 | 8.366 | 4.555 | 3.053, 2.947 |  |
| Q40 | 8.075 | 4.228 | 2.432, 2.246 | H^γ^, * 2.513, 2.513 ^##^ |
| V41 | 8.049 | 3.866 | 2.238 | H^γ1^, * 1.099; H^γ2^, * 1.004 |
| E42 | 8.264 | 4.211 | 2.512, 2.206 | H^γ^, * 2.639, 2.639 |
| D43 | 8.21 | 4.623 | 3.3 |  |
| L44 | 8.004 | 4.273 | 1.876, 1.826 | H^γ^, * 1.665; H^δ1^, * 0.944; H^δ2^, * 0.944 |
| N45 | 8.14 | 4.635 | 2.882, 2.882 | N^δ2^, * 7.414, 6.616 |
| L46 | 8.157 | 4.204 | 1.792, 1.792 | H^γ^, * 1.6; H^δ1^, * 0.97; H^δ2^, * 0.899 |
| D47 | 8.264 | 4.532 | 2.973, 2.939 |  |
| S48 | 7.949 | 4.328 | 3.991, 3.947 |  |
| L49 | 7.739 | 4.216 | 1.667, 1.667 | H^γ^, * 1.484; H^δ1^, * 0.902; H^δ2^, * 0.822 |
| W50 | 7.693 | 4.692 | 3.368, 3.368 | H^δ1^, * 7.266; H^ε3^, * 7.639; H^η2^, * 7.217; H^ζ2^, * 7.463; H^ζ3^, * 7.15; N^ε1^, * 9.685 |
| E51 | 7.761 | 4.25 | 2.113, 1.941 | H^γ^, * 2.31, 2.31 |

Footnotes: ^##^ The Hε21 and Hε22 resonances of Gln residues were found at 7.566-7.357 and 6.741-6.618 ppm, respectively, but were not assigned specifically.

^§§^ The Hβ2, Hβ3 and Hγ resonances of Leu-26 could not be determined due to signal overlap.

**HPLC and MS data for the full length and N- and C-terminal fragments of p9.**

Fig. 1: Quality analysis of *s*p^1-51^: A. HPLC chromatogram and B. MS spectrum

Fig. 2: Quality analysis of *s*p^1-21^: A. HPLC chromatogram and B. MS spectrum

Fig. 3: Quality analysis of *s*p^22-51^: A. HPLC chromatogram and B. MS spectrum
